# Phenotypic chemical screening using a zebrafish neural crest EMT reporter identifies retinoic acid as an inhibitor of epithelial morphogenesis

**DOI:** 10.1242/dmm.021790

**Published:** 2016-04-01

**Authors:** Laura Jimenez, Jindong Wang, Monique A. Morrison, Clifford Whatcott, Katherine K. Soh, Steven Warner, David Bearss, Cicely A. Jette, Rodney A. Stewart

**Affiliations:** 1 Department of Oncological Sciences, Huntsman Cancer Institute, University of Utah, Salt Lake City, UT 84112, USA; 2 Tolero Pharmaceuticals, Inc., Lehi, UT 84043, USA

**Keywords:** Drug screen, EMT, Epithelial mesenchymal, Neural crest, Retinoic acid, Zebrafish

## Abstract

The epithelial-to-mesenchymal transition (EMT) is a highly conserved morphogenetic program essential for embryogenesis, regeneration and cancer metastasis. In cancer cells, EMT also triggers cellular reprogramming and chemoresistance, which underlie disease relapse and decreased survival. Hence, identifying compounds that block EMT is essential to prevent or eradicate disseminated tumor cells. Here, we establish a whole-animal-based EMT reporter in zebrafish for rapid drug screening, called *Tg(snai1b:GFP)*, which labels epithelial cells undergoing EMT to produce *sox10*-positive neural crest (NC) cells. Time-lapse and lineage analysis of *Tg(snai1b:GFP)* embryos reveal that cranial NC cells delaminate from two regions: an early population delaminates adjacent to the neural plate, whereas a later population delaminates from within the dorsal neural tube. Treating *Tg(snai1b:GFP)* embryos with candidate small-molecule EMT-inhibiting compounds identified TP-0903, a multi-kinase inhibitor that blocked cranial NC cell delamination in both the lateral and medial populations. RNA sequencing (RNA-Seq) analysis and chemical rescue experiments show that TP-0903 acts through stimulating retinoic acid (RA) biosynthesis and RA-dependent transcription. These studies identify TP-0903 as a new therapeutic for activating RA *in vivo* and raise the possibility that RA-dependent inhibition of EMT contributes to its prior success in eliminating disseminated cancer cells.

## Abstract (precis)

**Editors' choice:** Generation and characterization of a novel neural crest EMT reporter for rapid *in vivo* drug screening in zebrafish that identifies a small-molecule EMT inhibitor that blocks this process by activating retinoic acid signaling.

## INTRODUCTION

The conversion of epithelial cells into migratory, invasive mesenchymal cells is a fundamental morphogenetic process during development and regeneration. Induction of epithelial-to-mesenchymal transition (EMT) causes epithelial cells to lose intracellular junctions and apical-basal polarity, and gain cytoskeleton reorganization, which are prerequisites for motility and invasion through surrounding tissue (Thiery et al., 2009). In addition, several studies have demonstrated that reactivation of developmental EMT programs in cancer cells constitutes a key step during metastasis (Thiery et al., 2009). EMT can endow cancer cells with pro-invasive properties to allow dissemination from the primary tumor and promote the acquisition of stem-cell-like properties (Mani et al., 2008), therapeutic resistance (Kurrey et al., 2009; Li et al., 2009), increased survival and immune-suppression (Polyak and Weinberg, 2009); all of which contribute to poor patient prognosis. For these reasons, targeting EMT in cancer patients has gained substantial therapeutic interest.

Our understanding of the cellular and molecular pathways controlling EMT in normal or cancerous cells remains incomplete, hindering efforts to rationally target EMT in the clinic. Indeed, most current small-molecule EMT inhibitors were discovered through unbiased cell-based *in vitro* screening techniques (Davis et al., 2014). However, these assays are usually restricted to single homogenous cell types and do not fully recapitulate the complex physiological environment in which other cell types and different extracellular matrix (ECM) components or ECM density impact EMT induction. Establishing *in vivo* EMT reporter assays for rapid screening are essential to complement conventional cell-based assays to identify the most effective EMT inhibitors for human disease. In addition, whole-animal-based EMT reporter models allow direct assessment of the effects of compounds on normal cell populations to determine tissue-specific toxicities, as well as to discover novel molecular pathways controlling physiological EMT that can be rationally targeted.

The embryonic dorsal neural tube of vertebrates is an excellent system in which to identify mechanisms controlling EMT because these cells undergo precisely timed and predictable EMT movements to form neural crest (NC) cells that migrate collectively or individually to generate a variety of cell types, such as cardiac, craniofacial and pigment cells, as well as neurons and glia of the peripheral nervous system (Green et al., 2015). Thus, defects in dorsal neural tube morphogenesis, EMT and NC cell migration underlie a number of human congenital diseases, particularly craniofacial abnormalities (Trainor, 2010). NC-derived lineages are also the origin of some of the most highly metastatic human cancers, such as melanoma and neuroblastoma, suggesting that these cancers have inherent or poised EMT and cell-migration mechanisms that allow rapid tumor dissemination. Indeed, seminal work that linked EMT to cancer metastasis showed that genes that are expressed during NC EMT are aberrantly activated during metastasis (Gupta and Massague, 2006; Kang and Massague, 2004; Yang et al., 2004). Among these are members of the Snail and Twist family of transcription factor genes, which repress the expression of epithelial cell adhesion molecules, including E-cadherin, to promote EMT during both development and in metastatic tumors (Gupta et al., 2005). This suggests that inhibitors of conserved signaling pathways controlling NC EMT will also be excellent therapies for blocking EMT during tumor invasion and/or metastasis.

Based on numerous studies in different vertebrate species, current models suggest that EMT in the dorsal neuroepithelium is induced by the combined actions of a number of growth factors secreted from the epiblast (BMP antagonists), underlying paraxial mesoderm (FGF) and ectoderm (Wnt) (Green et al., 2015). These pathways converge at the epithelial neural folds to induce the expression of canonical EMT transcription factors, such as *Snail1/2*, *Twist1* and *Zeb1/2*, as well as the NC ‘specifier’ genes *Sox9/10*, *Foxd3* and *tfAP2α* (Green et al., 2015). Thus, a gene regulatory network, mediated primarily through TGFβ/BMP and Wnt signaling, is proposed to control Snail1/2 and Twist1 expression and/or stability, which in turn promotes EMT to produce cells expressing NC specifier genes, such as *Sox10* (Simoes-Costa and Bronner, 2015). However, there is a large gap in our knowledge of how and where various growth factors directly induce canonical EMT transcription factor expression in NC progenitors and whether one or more of these factors are necessary for EMT. Indeed, to our knowledge, there are no examples in which a single pathway can inhibit epithelial morphogenesis to cause NC progenitors to remain trapped within the neural tube. In addition, the origin of the cranial NC has come under renewed scrutiny from recent studies in chick and mice that show NC-derived ectomesenchymal derivatives, such as cartilage, first arise (delaminate) from non-neural ectoderm adjacent to the neural folds, whereas a later population delaminates from within the neural tube (Breau et al., 2008; Lee et al., 2013a,b; Weston and Thiery, 2015). These studies suggest that different growth factor pathways might control NC EMT at different locations or times during cranial NC development. Identifying where and when essential, non-redundant growth factors and signaling pathways are required for NC EMT *in vivo* will be essential to understand NC development and NC-derived human diseases and cancers, as well as guide the rational design of EMT inhibitors *in vivo*.

Here, we develop a novel zebrafish EMT reporter called *Tg(snai1b:GFP)* that fluorescently labels neuroepithelial cells before NC specifier genes are expressed, allowing epithelial morphogenesis to be directly observed independent of cell-migration behaviors. Time-lapse and lineage tracing with *Tg(snai1b:GFP)* and the previously established *Tg(sox10:mRFP)* NC reporter line demonstrates that the zebrafish cranial NC delaminates initially from ectoderm overlying or adjacent to the neural keel, and then at later stages from cells within the neural rod, consistent with recent findings in mice and chick (Breau et al., 2008; Lee et al., 2013a,b; Weston and Thiery, 2015). Thus, the *Tg(snai1b:GFP)* strain can be used in whole-animal-based screening assays to identify genetic or chemical inhibitors of NC EMT at different developmental stages without the need to manually inject DNA constructs into individual embryos (Berndt et al., 2008). Indeed, we demonstrate that *Tg(snai1b:GFP)* is readily amenable to chemical genetic screening and identified a multi-kinase inhibitor, called TP-0903, that potently blocks EMT and cell migration in both delaminating cranial NC cell populations. Genomic analysis and chemical rescue experiments showed that TP-0903 activates retinoic acid (RA)-dependent signaling and transcription by increasing RA biosynthesis. Our findings reveal dual cellular origins for the cranial NC in zebrafish and unexpected roles for RA in inhibiting epithelial morphogenesis in these cranial NC progenitors. Finally, these studies suggest that activation of RA signaling through TP-0903 represents a new therapeutic strategy to inhibit EMT and eliminate residual metastatic disease.

## RESULTS

### Generation of the NC EMT reporter line: *Tg(snai1b:GFP)*

**Fig. 1.**
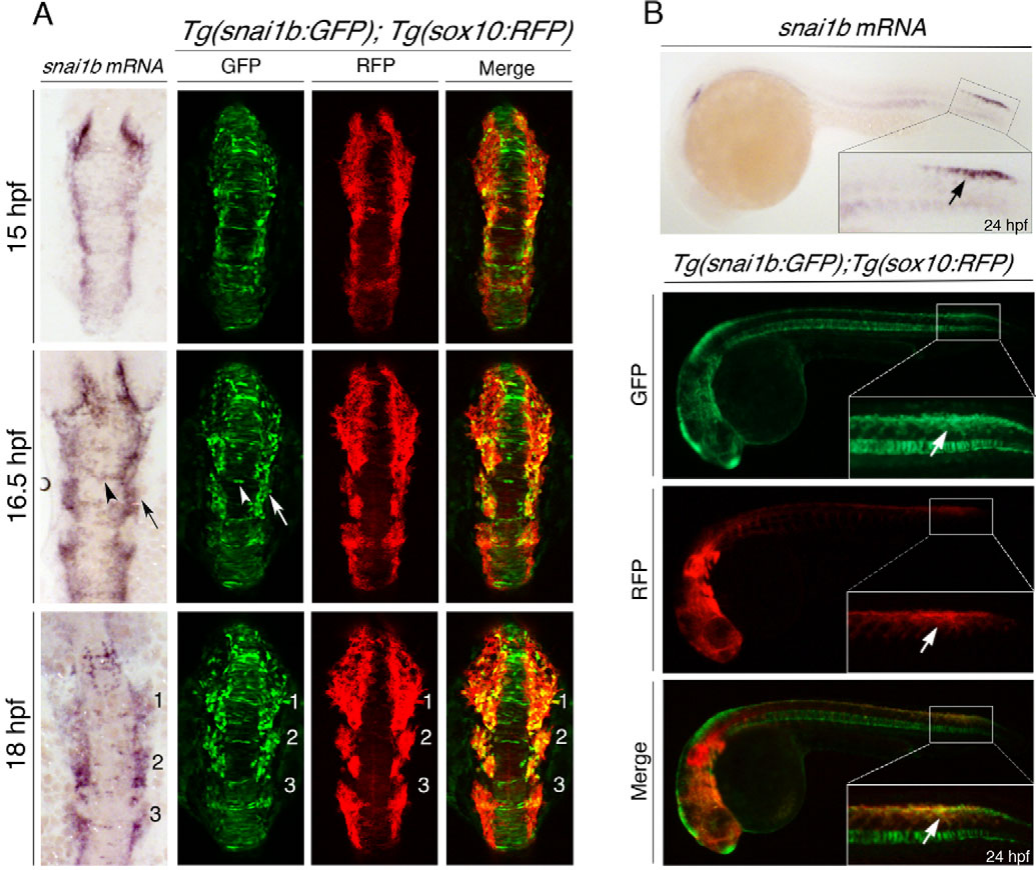
**Generating a NC EMT reporter during development.** (A) Dorsal views of whole-mount *in situ* hybridization of *snail1b* mRNA expression (left) compared to maximal *z*-projections of double-transgenic *Tg(snai1b:GFP); Tg(sox10:RFP)* zebrafish embryos (right) at the indicated developmental time points. *Tg(snai1b:GFP)* expresses GFP in the neuroepithelium (white arrowhead) and neural plate border cells (white arrow), similar to endogenous *snail1b* mRNA expression (black arrow and arrowhead). Comparison of *Tg(snai1b:GFP)* with *Tg(sox10:RFP)* shows that GFP is expressed in both the neuroepithelium and lateral plate border, whereas RFP labels delaminating NC cells at the neural plate border, partially overlapping with GFP (yellow cells). In migrating NC cells, GFP and RFP are co-expressed in the first two cranial streams (numbered). (B) Lateral views of brightfield (top) or epifluorescent (bottom) images of 24-hpf embryos comparing *snail1b* mRNA *in situ* hybridization expression in the trunk NC and *Tg(snai1b:GFP); Tg(sox10:RFP)* embryos, showing that *Tg(snai1b:GFP)* labels the same region as endogenous *snail1b* mRNA and co-labels with *sox10:RFP* in trunk NC cells (white arrows).

Snail transcription factors are inducers of EMT in a number of cell populations during metazoan development (Nieto, 2002). In zebrafish, the *snai1b* gene is expressed during early gastrulation in nascent mesodermal cells and later in pre- and post-migratory NC cells (Blanco et al., 2007 and Fig. 1A). To directly visualize epithelial morphogenesis during NC development, we isolated and sub-cloned approximately 3 kb of the *snai1b* promoter into a modified pEGFP-1 plasmid and generated a transgenic strain called *Tg(snai1b:GFP)* (see Materials and Methods). To determine the extent to which the *Tg(snai1b:GFP)* line recapitulates endogenous *snail1b* mRNA expression during NC development, we compared the fluorescent GFP pattern in *Tg(snai1b:GFP)* embryos to *snail1b* mRNA by fluorescent *in situ* hybridization at various development stages (Fig. 1 and Fig. S1). At 15 h post-fertilization (hpf), *Tg(snai1b:GFP)* embryos expressed GFP in neuroepithelial and ectodermal cells at the neural plate border, consistent with the endogenous *snail1b* mRNA expression pattern (Fig. 1A and Movie 1). Fluorescent *in situ* hybridization confirmed that endogenous *snai1b* mRNA colocalized with GFP-positive cells within presumptive NC cells at 15 hpf (Fig. S1A). This analysis also revealed that GFP levels became higher as cells adopted mesenchymal morphologies, while at the same time *snai1b* mRNA levels decreased, consistent with *snai1b* mRNA being rapidly downregulated in migrating cranial NC cells (see Fig. 1B and Fig. S1A, asterisk). We found that GFP protein levels perdured in migratory NC cells until ∼36 hpf, which allows the *Tg(snai1b:GFP)* reporter to be used as a short-term lineage marker for the NC (see below) and visualize cells before obvious EMT morphologies are observed.

At 16.5 hpf, when cranial NC streams migrate, GFP-positive cells appeared in migratory NC streams. This trend continued at 18 hpf, with GFP expression most prominent in the first and second cranial NC streams and most anterior neuroepithelium, whereas there was comparatively less GFP expression in the vagal NC stream, similar to the endogenous *snai1b* mRNA pattern (Fig. 1). At 24 hpf, GFP expression was diminished in most cranial NC cells and instead was expressed in delaminating trunk cells (Fig. 1B). We also observed GFP fluorescence in other tissue types in *Tg(snai1b:GFP)* embryos at later stages of development, such as the notochord and somites, which could reflect off-target and/or endogenous *snai1b* expression in these tissues, respectively (Fig. S1 and data not shown). The extent of GFP overlap with endogenous *snai1b* in these tissues was not examined further. Instead, we focused our efforts on characterizing the *Tg(snai1b:GFP)* strain as a novel reporter for NC cells undergoing EMT.

Previous studies show that *sox10*-promoter-driven fluorophores label cells undergoing delamination behaviors to produce NC cells (Berndt et al., 2008). To determine whether *Tg(snai1b:GFP)* embryos express GFP in the same cells as *sox10-*promoter-driven fluorophores, we generated double-transgenic *Tg(snai1b:GFP); Tg(sox10:RFP)* animals and analyzed EMT by two-color confocal time-lapse imaging during cranial NC development (Movie 2). Unexpectedly, we found that only *Tg(snai1b:GFP)* readily labeled midline neuroepithelial cells from 15- to 18-hpf (Fig. 1A), whereas *Tg(sox10:RFP)* more robustly labeled pre-migratory NC cells lateral to the neural rod (Fig. 1A). In migratory NC cells, both transgenes were expressed and partially overlapped, with *Tg(sox10:RFP)* being a more robust marker in this population, particularly in the vagal NC stream (Fig. 1A, Movie 2). Thus, the *Tg(snai1b:GFP)* transgenic line differs from previous NC reporters in its ability to selectively label epithelial progenitors within the neural keel/rod before EMT occurs, whereas *sox10*-driven transgenes preferentially label a lateral population of pre- and post-migrating cranial NC cells. The differences in the extent of overlap between GFP and RFP in the migrating NC cells in *Tg(snai1b:GFP); Tg(sox10:RFP)* animals might be due to differences in GFP protein stability in individual NC cells after *snai1b* transcription is normally downregulated during migration (see Fig. 1A and Fig. S1A) and/or due to the 3-kb *snai1b* promoter lacking some regulatory elements that would be required for expression of GFP in the lateral NC cell population, especially in the vagal NC stream.

### Visualization of EMT in *Tg(snai1b:GFP)* animals

**Fig. 2.**
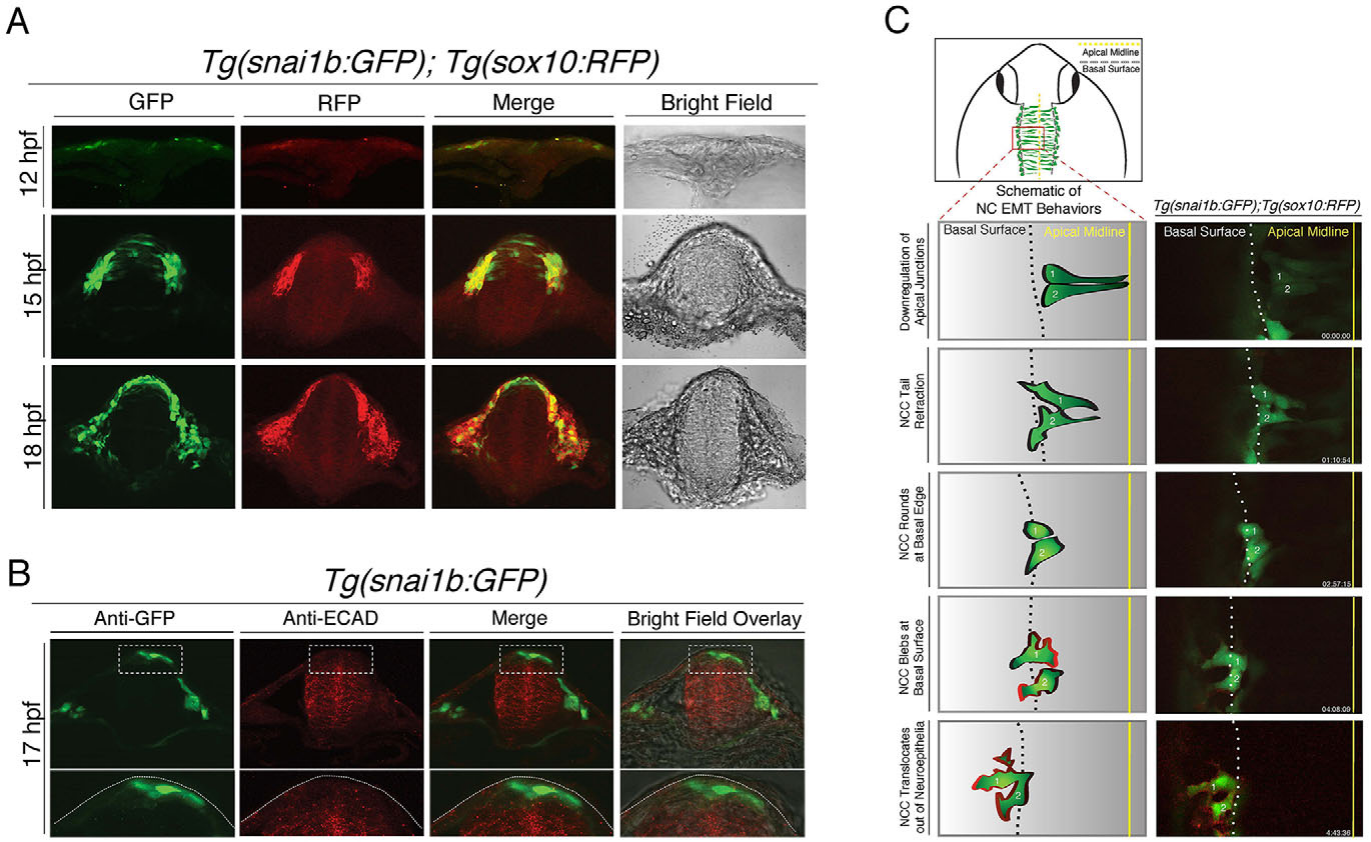
***Tg(snai1b:GFP)* labels dorsal neural tube progenitors that display morphological cell behaviors of EMT.** (A) Transverse hindbrain sections in double-transgenic *Tg(snai1b:GFP); Tg(sox10:RFP)* embryos at 12, 15 and 18 hpf (single confocal *z* plane, 40×) showing that *snai1b*-driven GFP is expressed in dorsal neural tube progenitor cells at 15 hpf, whereas both GFP and *sox10*-driven RFP are expressed in cells adjacent to the neural tube and migrating NC cells. (B) Transverse hindbrain sections of *Tg(snai1b:GFP)* embryos at 17 hpf processed for immunofluorescence analysis of Cdh1 (ECAD) and GFP. Bottom row displays higher-magnification views of boxed regions, and show decreased ECAD levels in GFP-positive cells compared to ventral neural tube. Dotted lines in the bottom panel outline the dorsal neural tube. (C) Schematic illustrating the imaging area (top) and cell delamination behavior (bottom left) derived from confocal time-lapse images of *Tg(snai1b:GFP); Tg(sox10:RFP)* embryos (bottom right), which captured two cells delaminating out of the neuroepithelium to produce *sox10*-positive NC cells (∼14 hpf to 18 hpf).

Our comparative study of *snai1b*- versus *sox10*-promoter-driven expression suggests that the *snai1b* promoter readily labels dorsal neuroepithelial cells with potential to undergo EMT and form NC cells (and does not label the entire neuroepithelium). To confirm this, we analyzed transverse sections through the hindbrain region at the level of rhombomere 4 of double-transgenic *Tg(snai1b:GFP); Tg(sox10:RFP)* animals at different developmental stages (Fig. 2A). GFP protein was first detectable at the neural keel stage (12 hpf) in cells overlying and adjacent to the keel but not within the neuroepithelial. At the neural rod stage (15 hpf), GFP was expressed in two distinct locations, within the dorsal half of the neural rod and in cells outside and lateral to the neural rod, whereas *sox10*-driven mRFP is expressed predominantly in cells outside the neural rod, overlapping with a subset of *snai1b*-GFP-expressing cells (Fig. 2A). At 18 hpf, GFP expression was restricted to the dorsal-most region of the neural tube and labeled a subset of *sox10*-positive migratory NC cells. In contrast, *sox10*-driven RFP was expressed robustly in migratory NC cells at 18 hpf, but remained absent from the dorsal neural tube (Fig. 2A). These studies suggest that cranial NC cells in zebrafish, like chick and mice, have dual origins that are spatially and temporally distinct, with the earliest delaminating population originating lateral and/or overlying the neural keel, followed by a second population emerging from within the dorsal neural rod and tube.

**Fig. 3.**
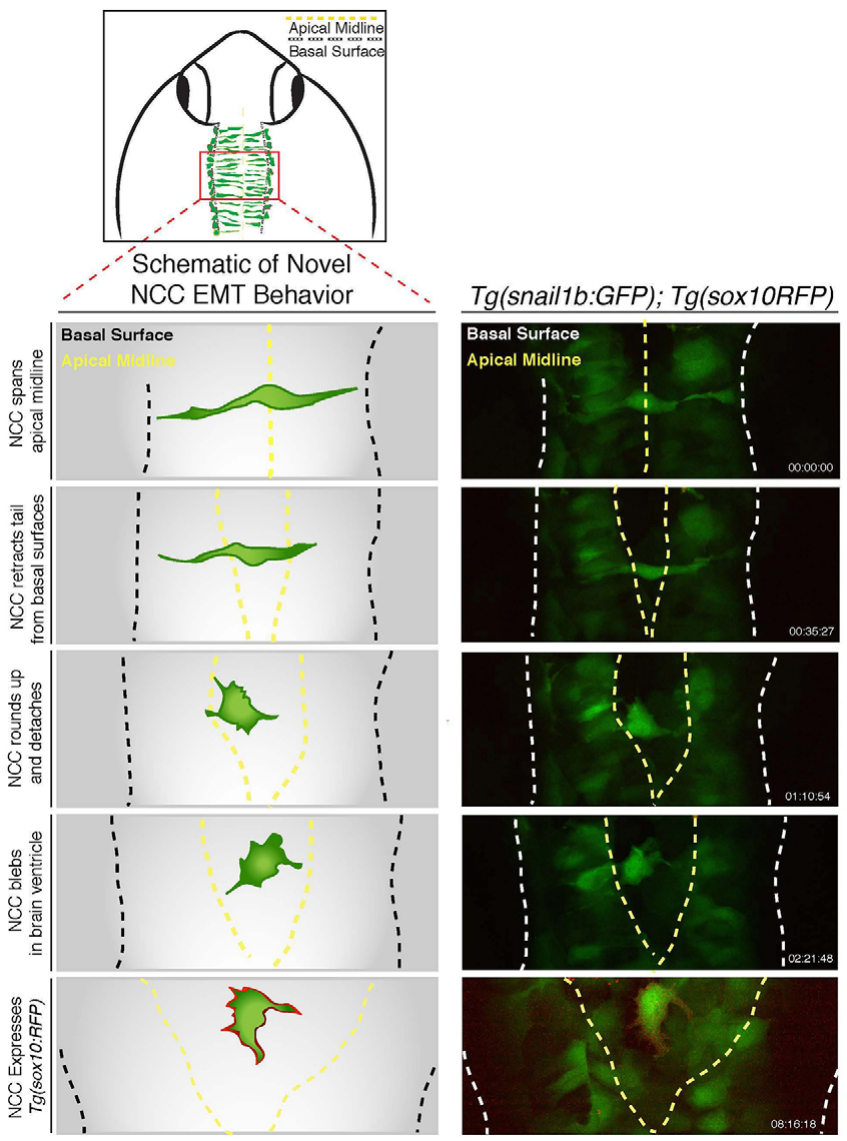
**Dorsal midline neural epithelial progenitor cells display novel** **delamination behaviors of EMT.** Schematic illustrating imaging area (top) and novel delamination behavior of a dorsal midline neuroepithelial progenitor (left); the schematic is derived from confocal time-lapse images of *Tg(snai1b:GFP); Tg(sox10:RFP)* embryos (right). Images show a GFP-positive cell initially spanning the dorsal midline before contracting apical attachments from each side of the neuroepithelium. Subsequently, GFP-positive cells detach and become rounded with filopodia and blebbing protrusions, and ultimately express *sox10-mRFP* at cell membranes. See also Movie 3.

The dorsal and transverse views of *Tg(snai1b:GFP)* animals suggest that the GFP-positive cells are fated to undergo an EMT and become NC cells. To confirm this, we first analyzed the expression of E-cadherin protein, whose loss is a hallmark of EMT, in transverse views of *Tg(snai1b:GFP)* animals, and show that dorsally restricted GFP-positive cells express reduced levels of E-cadherin compared to the rest of the neural tube (Fig. 2B). We next analyzed time-lapse recordings of double-transgenic *Tg(snai1b:GFP); Tg(sox10:RFP)* animals to follow individual cell behaviors as cells emerge from the neural tube (Fig. 2C and Movie 3). Confocal time-lapse analysis using GFP as a short-term lineage tracer confirmed that GFP-positive neuroepithelial cells in *Tg(snai1b:GFP)* animals exhibit EMT behaviors to emerge as *sox10*-positive NC cells (Fig. 2C). We did not lineage-trace *snai1b*-GFP-positive cells in other axial regions arising from the neural rod (anterior forebrain or trunk), so we cannot rule out the possibility that a subset of GFP-positive cells also generate other lineages in these locations. Nonetheless, in the hindbrain region we did observe that subsets of neuroepithelial cells contact the apical midline and basal surface. As cells initiate EMT they detach from the apical midline by downregulating adherens junctions and retract their apical tail. Subsequently, epithelial cells round up near the basal surface and use blebbing motility to translocate out of the neural tube, ultimately gaining directed motility away from the neural tube (Fig. 2C schematic). The same cellular behaviors were originally described from live-imaging studies of *sox10*-positive NC cells undergoing EMT (Berndt et al., 2008; Clay and Halloran, 2013). Confocal time-lapse analysis of the delaminating dorsal midline population at later developmental stages in *Tg(snai1b:GFP); Tg(sox10:RFP)* animals revealed previously unidentified cellular behaviors of the dorsal-most neural tube cells (Fig. 3 and Movie 4), which span across the apical midline to contact both sides of the neuroepithelium. During delamination, these cells retract from both sides of the neuroepithelium simultaneously and lose polarity to become rounded. Subsequently, these cells flatten and extend cellular protrusions, such as filopodia and blebs, and begin to express the NC marker *sox10:RFP* (Fig. 3). These studies show that the *Tg(snai1b:GFP)* line labels two delaminating populations of neuroepithelial cells that become NC cells, and represents a novel whole-animal EMT reporter line that can be used for chemical screening.

### Chemical screening approach to identify EMT inhibitors *in vivo*

Genetic crosses of the *Tg(snai1b:GFP)* line can generate thousands of embryos per day, allowing rapid and direct visualization of EMT behaviors *in vivo* after genetic or chemical perturbations. To determine whether the *Tg(snai1b:GFP)* line could be used to identify compounds that block NC EMT *in vivo*, we performed a pilot chemical screen. *Tg(snai1b:GFP)* embryos were treated with different doses of kinase inhibitors (ranging from 0.1 to 100 µM) at the 3- to 8-somite stage (∼13 hpf), time points that (1) avoid developmental delays due to interfering with gastrulation movements and (2) allow growth factor induction to occur at the neural plate border to induce EMT factors, but (3) precedes the onset of EMT in cranial regions. Embryos were treated for 6-12 h and then visualized for GFP fluorescence in the neural tube (see Materials and Methods and Fig. S2). Failure of cells to undergo EMT was determined by visualizing the accumulation of GFP-positive cells within the dorsal neural tube. Surprisingly, under our assay conditions, most chemical compounds did not cause overt epithelial morphogenesis phenotypes *in vivo* (Table S1), including those that target kinase and cytoskeletal remodeling pathways, such as the MAPK/ERK, PI3K/AKT and Rho GTPase pathways, previously shown to affect individual cell behaviors during EMT *in vitro* or *in vivo* (Berndt et al., 2008; Irie et al., 2005; Zheng et al., 2013)*.* These results suggest that multiple signaling pathways might compensate for each other to drive the initial epithelial morphogenesis stages of the EMT program during NC development (see Discussion).

**Fig. 4.**
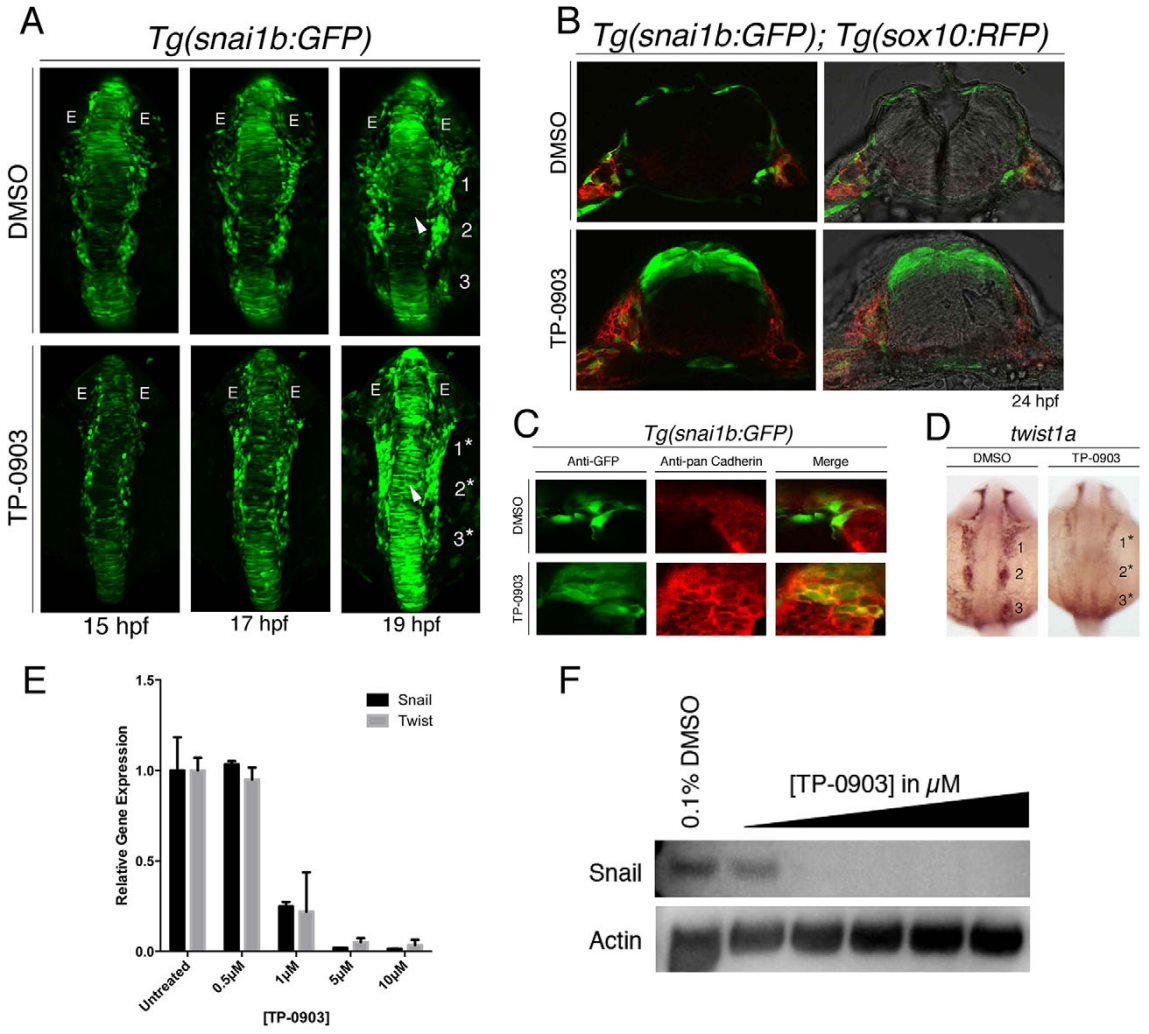
**Chemical screening with *Tg(snai1b:GFP)* identifies TP-0903 as an inhibitor of NC EMT.** (A) Dorsal views of *Tg(snai1b:GFP)* embryos treated with DMSO (top) or TP-0903 (bottom) at 11.5 hpf and imaged at the indicated time points by 10× confocal time-lapse microscopy. TP-0903-treated embryos accumulate GFP-positive cells in the dorsal neural tube (arrowhead) and lateral cells remain associated with the neural plate border and are fused (19/20 embryos) (compare numbered streams in DMSO control to numbered asterisks in TP-0903). E, eye. (B) Transverse sections of DMSO (top)- and TP-0903-treated (bottom) *Tg(snai1b:GFP); Tg(sox10:RFP)* embryos at 24 hpf, showing that TP-0903 causes accumulation of GFP-positive cells in the dorsal neural tube (4/5 embryos). (C) Transverse sections through hindbrain of *Tg(snai1b:GFP)* embryos at 24 hpf treated with DMSO (top) or TP-0903 (bottom) and analyzed by immunofluorescence for pan-Cadherin and GFP, showing that GFP-positive cells in TP-0903-treated embryos maintain Cadherin levels and cell-cell adhesion within the neural tube (3/4 embryos). (D) Dorsal views of DMSO (left)- and TP-0903-treated (right) embryos at 18 hpf processed for *twist1a* mRNA *in situ* hybridization, showing that TP-0903 significantly reduces *twist1a* expression levels (18/20 embryos) (compare numbered streams in DMSO to numbered asterisks in TP-0903). (E) Analysis of *TWIST1* and *SNAIL1* mRNA expression in DMSO- and TP-0903-treated PANC-1 cells by RT-qPCR. Two-way ANOVA was used to measure statistical significance. (F) Western blot analysis of PANC-1 cells treated with DMSO or 0.5-10 µM TP-0903, showing a dose-dependent decrease in SNAIL1 protein expression.

Although compounds that primarily target one major signaling pathway did not show a significant effect on NC EMT, a multi-kinase inhibitor, called TP-0903 [formally known as Compound 13 (Mollard et al., 2011)], dramatically blocked EMT and NC cell migration (Fig. 4). Confocal time-lapse analysis of *Tg(snai1b:GFP)* embryos treated with TP-0903 between 11 and 19 hpf showed significant accumulation of GFP-positive cells within the developing neuroepithelium (Fig. 4A, compare Movies 5 and 6). In addition, TP-0903 treatment of *Tg(snai1b:GFP)* embryos halted migration of NC cells that had already exited the neural tube and fused the first two cranial NC streams together (Fig. 4A). Differentiation of lens placodes, somites or other NC-derived tissues themselves (such as melanized pigment cells; see later) showed that these tissues formed at the appropriate developmental stages, suggesting that TP-0903 did not cause a general developmental arrest.

To confirm that TP-0903 treatment inhibited EMT, we analyzed cross-sections of *Tg(snai1b:GFP); Tg(sox10:RFP)* embryos treated with TP-0903 or DMSO. In DMSO-treated embryos, the majority of the GFP-positive cells had delaminated from the neural tube by 24 hpf to form *sox10*-positive NC cells. In contrast, TP-0903-treated embryos retained GFP-positive progenitor cells in the dorsal neural tube (Fig. 4B). Analysis of Cadherin expression in the TP-0903-treated embryos showed that GFP-positive cells expressed elevated Cadherin levels compared to DMSO-treated controls (Fig. 4C). Finally, to determine whether TP-0903 directly affects the expression of canonical EMT transcription factors, we analyzed expression of *snai1b*,* twist1a* and *zeb1a* by RNA *in situ* hybridization (Fig. 4D). We found that TP-0903 caused an almost complete loss of *twist1a* expression in cranial NC cells at 18 hpf, whereas the expression of other EMT transcription factors was not significantly affected (Fig. 4D and data not shown). Consistent with our *in vivo* findings, TP-0903 reversed the mesenchymal features of human cancer cells (Fig. 4E,F), showing that TP-0903 is also a potent EMT inhibitor *in vitro*. Thus, the *Tg(snai1b:GFP)* transgenic strain is an effective whole-animal-based model for identifying conserved EMT and cell-migration inhibitors, and identifies TP-0903 as a potent EMT inhibitor.

### TP-0903 induces a rapid retinoic acid transcriptional response

**Fig. 5.**
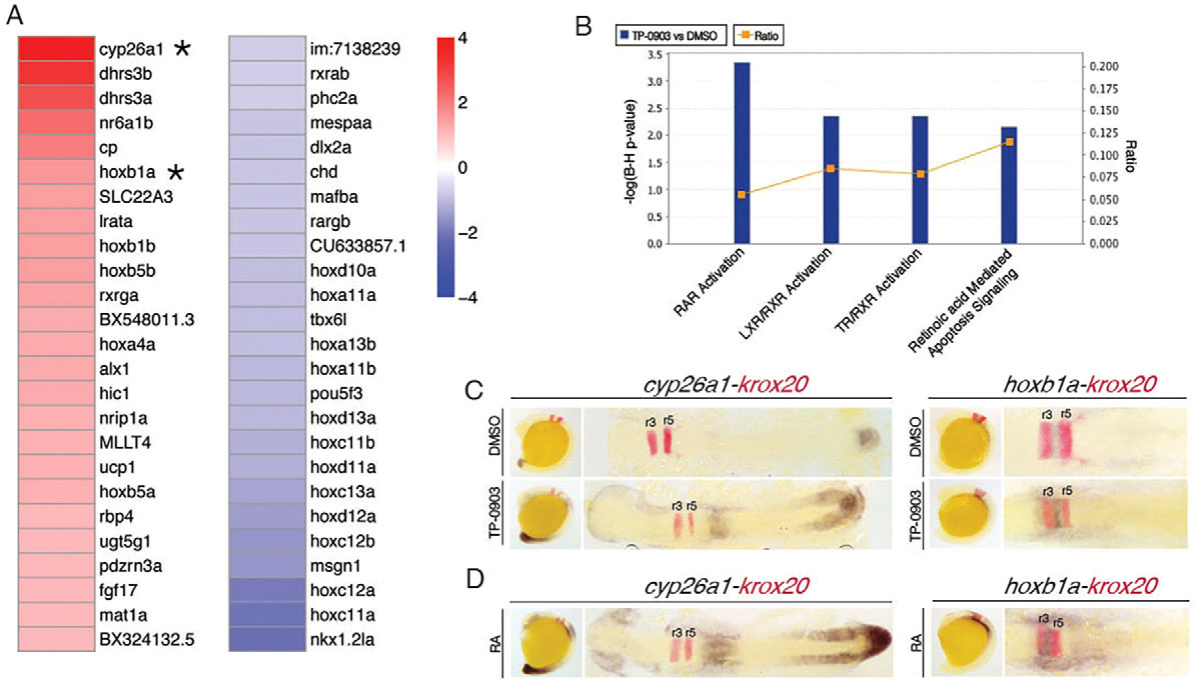
**TP-0903 acts by inducing retinoic acid signaling in zebrafish embryos.** (A) Heat map schematic showing the 50 most differentially expressed transcripts from RNA-Seq as measured by log2 ratio from 4-h-treated DMSO and TP-0903 embryos. Asterisks indicate transcriptional targets of RA examined by whole-mount *in situ* hybridization in TP-0903-treated embryos. (B) Ingenuity pathway analysis of differentially expressed genes in TP-0903-treated embryos reveals that RA signaling pathways are exclusively activated. Bars represent the *P*-value for each RA pathway (expressed as −1×log of the *P*-value). The yellow line represents the ratio of the number of genes from our data set represented within each pathway to the total number of genes in each pathway. (C) Lateral (left panels) and dorsal (right panels) flat-mounted views of *in situ* hybridization at 16 hpf for two genes, *cyp26a1* and *hoxb1a*, identified as elevated by RNA-Seq after 4 h TP-0903 treatment. In all panels, *krox20* (red) is used as a marker of rhombomeres r3 and r5 (red stain). (D) Lateral (left panels) and dorsal (right panels) flat-mounted views of embryos treated at 13 hpf with RA mimic TP-0903 expression effects on *cyp26a1* and *hoxb1a*.

TP-0903 was originally designed to act as a competitive inhibitor of the AXL receptor tyrosine kinase (IC_50_=27 nmol/l; Mollard et al., 2011). However, TP-0903 also shows significant activity (>80%) against at least 11 other kinases, including the three TAM family members (AXL, MER and TYRO3) as well as Aurora A, JAK2, ALK and ABL1 (Mollard et al., 2011). In addition, our pilot screen showed that inhibiting single TP-0903 targets was insufficient to recapitulate the TP-0903 NC EMT phenotypes, suggesting that multiple pathways and/or unknown targets are inhibited by TP-0903 to block EMT. Importantly, the loss of EMT transcription factor expression, such as *twist1a*, within a few hours of treatment suggested that TP-0903 acts through a rapid transcriptional mechanism. Therefore, we performed RNA sequencing (RNA-Seq) analysis on DMSO- and TP-0903-treated embryos to determine whether TP-0903 elicits an immediate or delayed transcriptional response. We treated zebrafish embryos with TP-0903 or DMSO at the 8-somite stage (13 hpf), when the brain primordium has thickened into the neural keel and cranial neuroepithelial cells are commencing EMT. Embryos were treated for 1, 4 or 8 h and subsequently divided into two groups to (1) extract RNA for gene expression analysis or (2) fix cells for *in situ* hybridization to confirm differential expression of candidate genes identified from RNA-Seq in TP-0903-treated embryos (Fig. 5).

Analysis of the relative expression levels of transcripts in TP-0903- and DMSO-treated embryos at different time points showed that TP-0903 induces a rapid transcriptional response in embryos after just 1 h, which becomes more robust by 4-h post-treatment. Analysis of differentially expressed transcripts at 1 and 4 h identified several RA-target genes, including genes from within the retinoid pathway itself, such as *rxrga*, *dhrs3a/3b* and *cyp26a1*, and members of the Hox gene family that harbor RA response elements (RAREs), including *hoxb1a*, *hoxb5b* and *hoxa4a* (Fig. 5A). Ingenuity pathway analysis of genes differentially expressed in TP-0903-treated embryos at 4 h showed that RA pathway activation represented the top four canonical pathways affected by TP-0903 (Fig. 5B and Fig. S3). To validate our findings, we examined the expression of two differentially expressed genes, *cyp26a1* and *hoxb1a*, by whole-mount *in situ* hybridization in TP-0903-treated embryos. Both *cyp26a1* and *hoxb1a* are direct transcriptional targets of RA and *cyp26a1* is commonly used to report RA activity (Niederreither and Dolle, 2008). Our *in situ* hybridization analysis confirmed the RNA-Seq results and showed that the intensity of *cyp26a1* and *hoxb1a* expression was increased in TP-0903-treated embryos compared to controls at 16 hpf (Fig. 5C). We also treated embryos with RA at the 8-somite stage (13 hpf) and found that it mimicked TP-0903 treatment with respect to increased *cyp26a1* and *hoxb1a* expression, although with a more robust response (Fig. 5D).

**Fig. 6.**
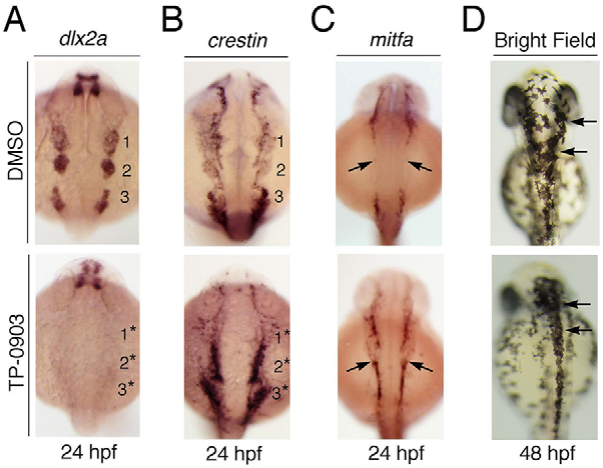
**TP-0903 inhibits expression of *dlx2a* and induces expression of *mitfa* in the presumptive ectomesenchymal NC cells.** Dorsal views of embryos treated with DMSO or TP-0903 at the 3-somite stage and fixed at the indicated time points for processing by whole-mount *in situ* hybridization and brightfield microscopy with the indicated probes or direct visualization of pigment cells. (A) Compared to controls (top panel), TP-0903 completely inhibits *dlx2a* expression in cranial NC streams (18/20 embryos). (B) Analysis of TP-0903-treated embryos with the pan-NC marker *crestin* shows that NC cells that normally express *dlx2a* lateral to the neural tube are induced but fail to migrate away, and the first two cranial NC streams are fused together, as indicated by numbered asterisks (13/18 embryos). (C) Analysis of melanophore precursor marker *mitfa* in control and TP-0903-treated embryos show that TP-0903 induces aberrant *mitfa* expression in the NC cells lateral to the neural tube (18/20 embryos) (black arrows). (D) Analysis of differentiated melanophores at 48 hpf shows that TP-0903 causes accumulation of melanophores on the dorsal neural tube (arrows), but not adjacent to the neural tube (18/21 embryos).

TP-0903 treatment induced an RA-like response, so we next analyzed the expression pattern of NC genes previously shown to be affected by RA treatment (Ellies et al., 1997; Lee et al., 1995; Matt et al., 2003). We analyzed the expression of *dlx2a* in TP-0903-treated embryos and found that it was inhibited, showing that TP-0903, like RA itself, inhibits ectomesenchymal differentiation of NC cells (Fig. 6A). Because previous studies did not follow the fate of these cells to determine whether RA blocked differentiation, induced apoptosis and/or induced trans-differentiation, we performed *in situ* hybridization analysis with the pan-NC marker *crestin*, which showed that the cranial NC cells in TP-0903-treated embryos were still present but the first two cranial NC streams were fused together adjacent to the neural tube (Fig. 6B), a phenotype also observed in *Tg(snai1b:GFP)* embryos treated with TP-0903 (Fig. 4A). Thus, cranial NC cells still form in TP-0903-treated embryos, but fail to express *dlx2a* or undergo normal collective migration behaviors.

To determine whether the lateral population of *crestin*-positive cells remains undifferentiated or has the potential to differentiate into other lineages, we analyzed *mitfa* expression, a marker for melanocyte precursors. TP-0903 treatment caused aberrant *mitfa* expression adjacent to the neural tube, the same region where *dlx2a* is lost (Fig. 6C, arrows). Thus, at the stages we analyzed, TP-0903 represses *dlx2a* and promotes the expression of *mitfa* in the NC cells adjacent to the neural tube. Analysis of differentiated melanophores at 48 hpf showed that TP-0903 did not cause accumulation of melanophores adjacent to the neural tube, the location where *mitfa* was aberrantly expressed at 24 hpf (Fig. 6D). This suggests that the lateral population of *dlx2a^−^/crestin^+^/mitfa^+^* NC cells observed at 24 hpf in TP-0903-treated embryos might differentiate into other lineages or undergo apoptosis by 48 hpf (see Discussion). Instead, TP-0903 caused accumulation of pigment cells on top of the neural tube, a region that normally produces melanophores, suggesting that TP-0903 inhibits cell migration in the dorsal/medial NC cell population (Fig. 6D, arrows). Together, these results show that TP-0903 causes a specific and rapid increase in RA-dependent transcription during cranial NC development, which in turn inhibits EMT and/or cell migration in the medial and lateral delaminating populations. We also show that TP-0903 inhibits differentiation of *dlx2a*-positive ectomesenchymal cells, which instead express the pan-NC marker *crestin* and the melanophore marker *mitfa*.

**Fig. 7.**
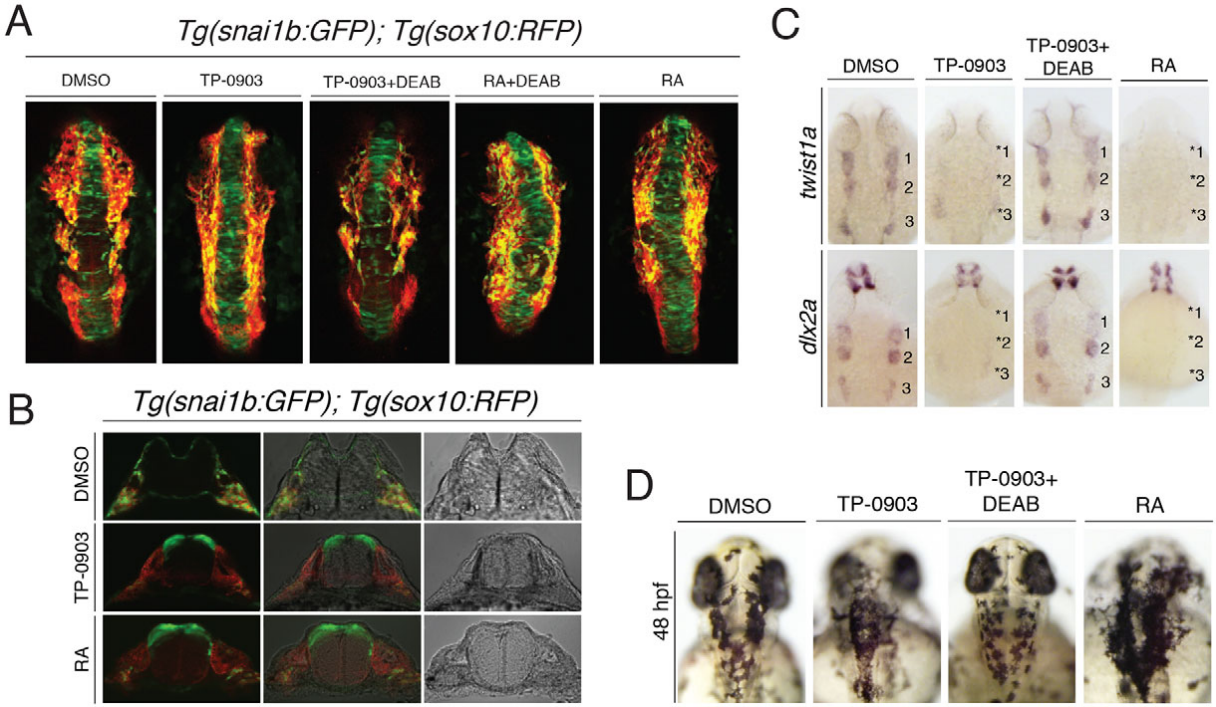
**Retinoic acid controls cranial NC EMT, cell migration and ectomesenchyme differentiation.** (A) Dorsal views of maximal *z*-projection confocal images of *Tg(snai1b:GFP); Tg(sox10:RFP)* embryos treated with the indicated compounds at 11.5 hpf. TP-0903 inhibits NC EMT and cell migration, which is rescued by co-treatment with DEAB (16/18 embryos), an inhibitor of retinaldehyde dehydrogenase (RALDH), which is required for RA biosynthesis. In contrast, direct treatment with RA bypasses the requirement for RALDH and is not rescued by DEAB (0/19 embryos). (B) Transverse sections through the hindbrain of *Tg(snai1b); Tg(sox10:RFP)* embryos at 24 hpf treated with DMSO, TP-0903 or RA confirms that RA treatment causes accumulation of GFP-positive dorsal neural tube cells similar to TP-0903 treatment (6/8 embryos). (C) Dorsal views of 24-hpf embryos processed by whole-mount *in situ* hybridization for *twist1a* and *dlx2a* mRNA, showing that inhibition of RA synthesis in TP-0903-treated embryos rescues expression of these genes in NC streams (numbered) (15/18 and 18/21 embryos, respectively), whereas RA treatment itself mimics TP-0903 and blocks expression of *twist1a* (14/16 embryos) and *dlx2a* (18/18 embryos)*.* (D) Dorsal views of 48-hpf embryos, showing that melanophores accumulate on the head of TP-0903-treated embryos (17/19 embryos), which is rescued by DEAB co-treatment (17/20 embryos) and mimicked by direct RA treatment (19/20 embryos).

To directly test whether TP-0903 acts through RA signaling, we inhibited RA synthesis in TP-0903-treated embryos by co-treating *Tg(snai1b:GFP); Tg(sox10:RFP)* embryos with TP-0903 and diethylaminobenzaldehyde (DEAB), a potent inhibitor of retinaldehyde dehydrogenase (RALDH), the rate-limiting enzyme required for the final conversion of retinal to RA. Inhibition of RA synthesis by DEAB in TP-0903-treated embryos rescues the NC EMT and cell-migration defects (Fig. 7A and compare Movies 2, 7 and 8). Co-treatment of TP-0903 and DEAB also rescued *twist1a* and *dlx2a* expression and pigment patterning defects caused by TP-0903 treatment (Figs 7C and 6D). As expected, DEAB did not rescue RA-treated embryos because exogenous RA treatment bypasses the need for Raldh2 and retinal conversion (Fig. 7A and Movie 9). These results confirm that TP-0903 is a novel small-molecule compound that can activate RA biosynthesis and transcriptional responses *in vivo*.

### Retinoic acid controls cranial NC EMT, cell migration and ectomesenchymal differentiation

The genomic, molecular and cellular analysis of TP-0903-treated embryos shows potential new roles for RA signaling in dorsal neural tube EMT. To directly test whether RA itself could inhibit EMT, we treated 3-somite stage (11.5 hpf) *Tg(snai1b:GFP); Tg(sox10:RFP)* embryos with RA and found that it causes an accumulation of GFP-positive cells in the neuroepithelium and RFP-positive NC cells along the neural plate border, mimicking the TP-0903 treatment (Fig. 7A,B; and Movie 10). Additionally, we also found that RA treatment inhibits expression of EMT regulator *twist1a* and chondrogenic differentiation marker *dlx2a*, and causes an accumulation of pigment cells on the neural tube at 48 hpf (Fig. 7C,D). These results demonstrate that increased RA is sufficient to impair NC EMT, cell migration and differentiation, and indicate that the NC phenotypes observed in TP-0903-treated embryos act through an RA-dependent transcriptional response. Thus, RA signaling controls multiple events during cranial neural tube development: (1) delamination of NC progenitors at the neural plate border and within the neural rod/tube, (2) collective cell migration away from the neural tube, and (3) differentiation of ectomesenchymal progenitors. Although some of these RA-dependent phenotypes have been observed previously during NC development, these data are the first to show that RA-dependent transcriptional responses directly inhibit cranial NC EMT *in vivo*.

## DISCUSSION

EMT is essential for embryogenesis, regeneration and cancer metastasis, and requires interactions with multiple cell types and ECM components (Lamouille et al., 2014). Modeling the physiological complexity of EMT is not feasible using conventional *in vitro* cell-based systems. Despite this shortcoming, most EMT inhibitors identified to date are discovered through cell-based assays, which likely contributes to the lack of effective EMT inhibitors in the clinic (Davis et al., 2014). In addition, our knowledge of the essential signaling pathways controlling EMT *in vivo* remains incomplete owing to difficulties in visualizing EMT in living animals. In this study we take advantage of the unique imaging and *ex utero* development attributes of zebrafish to develop an EMT reporter strain, *Tg(snai1b:GFP)*, to directly image EMT in intact animals and identify new *in vivo* inhibitors of this process. Through this approach we show that a multi-kinase inhibitor, called TP-0903, blocks EMT by activating RA signaling. Remarkably, despite numerous studies examining RA in hindbrain and NC development (Ellies et al., 1997; Lee et al., 1995; Maclean et al., 2009; Rhinn and Dolle, 2012; Uehara et al., 2007; Vieux-Rochas et al., 2010; White and Schilling, 2008), this is the first study showing that RA directly controls EMT in cranial NC progenitors. Finally, because RA is a well-established differentiation agent, these findings suggest that RA could antagonize EMT-dependent pathways controlling de-differentiation and stem-cell-like properties, providing an alternative explanation for the effectiveness of retinoids as adjuvant therapies to eliminate residual tumor cells in cancer patients.

Detailed characterization of the *Tg(snai1b:GFP)* strain showed that delaminating cranial NC cells are located in two distinct locations during neural tube morphogenesis: a lateral population located in presumptive non-neural ectoderm adjacent to or overlying the neural keel and a later delaminating population arising from dorsal neuroepithelial cells within the neural rod/tube (Figs 2 and 3). These findings are consistent with previous morphological and single-cell-labeling studies in zebrafish that suggested that cranial NC cell populations are laterally segregated from the neural keel and do not reach the dorsal midline at early stages (12 hpf), whereas, at later stages (16.5 hpf), cells within the neuroepithelium begin to delaminate to form migratory NC cells (Schilling and Kimmel, 1994). Thus, our studies provide additional evidence for a dual origin of the cranial NC that was recently described in mouse and chick embryos (Breau et al., 2008; Lee et al., 2013a,b; Weston and Thiery, 2015), and suggest that the genetic mechanisms controlling cranial NC cell delamination and ectomesenchymal specification are highly conserved from fish to mammals. Importantly, our live-imaging studies show that RA signaling is a critical negative regulator of delamination in both cranial NC cell populations. In addition, our results suggest that RA signaling needs to be repressed for ectomesenchymal cell fates to be established from the lateral population (Fig. 6). In the presence of RA or TP-0903, *dlx2a*-positive ectomesenchymal cells are lost and instead these cells express non-ectomesenchymal markers, such as *mitfa*, suggesting that RA-dependent transcription might promote an ectomesenchymal to non-ectomesenchymal fate switch. Future fate-mapping studies, including detailed analysis of differentiated cell types and/or apoptosis markers at later developmental stages, are needed to determine the extent to which RA induces such a fate change and/or promotes cell death in this lateral population.

A number of NC-specific transgenic strains in zebrafish have been established (Berndt et al., 2008; Gilmour et al., 2002; Hochgreb-Hagele and Bronner, 2013; Kirby et al., 2006). These strains robustly label pre- and post-migratory NC cells overlying or adjacent to the neural keel; however, to our knowledge, the *Tg(snai1b:GFP)* strain is unique in its ability to label the later dorsal/medial delaminating population, as demonstrated by our comparative analysis of *Tg(sox10:mRFP)* and *Tg(snai1b:GFP)* expression in Fig. 2. In addition, our time-lapse imaging studies show that GFP-positive neuroepithelial cells become *sox10*-positive NC cells, showing that the *Tg(snai1b:GFP)* strain is the earlier marker of the presumptive NC. Thus, the *Tg(snai1b:GFP)* line can be used to identify genetic and chemical perturbations that impact the initial stages of epithelial morphogenesis before more recognizable features of EMT are detected, such as membrane detachments, protrusive behaviors and cell migration. Indeed, previous studies that depend on these later EMT events could explain why RA was previously overlooked as an EMT inhibitor during NC development, despite extensive studies investigating the role of RA in hindbrain development. Conversely, because our screening assay was designed to identify inhibitors that caused accumulation of GFP-positive epithelial cells within the neural tube, most of the candidate EMT inhibitors that we screened did not overtly affect EMT, although some did inhibit NC cell migration. For example, if cell migration were the endpoint of our assay, FGF, JAK/STAT and RhoA pathway inhibitors would have been scored as EMT inhibitors *in vivo*. These results highlight the risk of relying on cell migration/mesenchymal features as the endpoint when screening for genes/inhibitors of EMT, because they might overlook molecules that disrupt other epithelial morphogenesis programs, such as epithelial-to-epithelial sheet or epithelial-to-amoeboid transitions, and/or the ‘metastable’ cellular reprogramming feature of EMT, which might be more therapeutically relevant. Importantly, the *Tg(snai1b:GFP)* line and assays that we have developed in this study allow us to perform unbiased screens for such inhibitors, which can be rapidly validated in human cancer cell models (Fig. 4). In addition, future studies testing combinations of *in vitro* EMT inhibitors in *Tg(snai1b:GFP)* will likely uncover compensatory/parallel signaling pathways required for EMT *in vivo*, guiding the use of inhibitor combinations in human disease and cancer.

Early developmental exposure to RA causes overt defects in cranial structures in humans and other vertebrates, particularly in the hindbrain region (Lammer et al., 1985; Lee et al., 1995). Isolated cranial NC cells are highly susceptible to developmental reprograming by RA (Williams et al., 2004) but the consequence of this reprogramming is not known. Interestingly, Cyp26c1, an enzyme that oxidizes and degrades RA to control levels of RA in the neural ectoderm (Abu-Abed et al., 2001; Emoto et al., 2005; Hernandez et al., 2007; Melton et al., 2004; Schilling et al., 2012; Trainor and Krumlauf, 2000; White and Schilling, 2008), was recently established as a newly identified target of neural-plate-border-specifier genes *Pax3* and *Zic1* (Plouhinec et al., 2014). Pax3 and Zic1 directly activate a NC gene regulatory network (*snail1*, *snail2*, *foxd3* and *twist1*) that is sufficient to promote NC cell specification (Milet et al., 2013; Plouhinec et al., 2014). Together with our data, these findings suggest a model in which Pax3 and Zic1 act to suppress RA signaling at the neural plate border while simultaneously activating *snail1* expression to induce EMT.

Our studies are the first to show that RA inhibits cranial NC EMT. There are several ways that RA could be blocking EMT. For example, RA might activate transcription of epithelial cell-cell adhesion molecules to prevent detachment from the neuroepithelium, as observed in TP-0903-treated embryos (Fig. 4C). Consistent with this, increased E-cadherin has been observed in NC cells that failed to emigrate from the neural tube in *Cyp26a1/c1^−/−^* mutant mice (Uehara et al., 2007). Additionally, RA treatment increases cytosolic calcium levels (Davis et al., 1991) and cell-cell adhesion of cultured NC cells (Smith-Thomas et al., 1987), and, in cancer cell lines, RA can activate E-cadherin expression and promote cadherin stabilization at cell membranes (Shah et al., 2002; Woo and Jang, 2012). These observations together with our data suggest that an excess of RA impairs NC EMT and the production of migratory NC cells by stimulating Cadherin expression and increasing localization at cell membranes. Future studies using the *Tg(snai1b:GFP)* line will allow us to isolate and purify dorsal neural tube progenitors by fluorescent-activated cell sorting (FACS) analysis to determine whether RA directly regulates Cadherin expression and/or identify other currently unknown RA-effector pathways that inhibit NC EMT.

Induction of EMT can generate cancer cells with stem-cell-like characteristics and contribute to the formation of poorly differentiated tumors with increased invasion and metastatic potential. Adjuvant therapies that target dormant/cancer stem cells are already incorporating RA-like molecules into differentiation therapy methods. In the clinic, retinoids are thought to act by promoting an arrest in cell proliferation, inducing differentiation and subsequent cell death (Nasr et al., 2008; Reynolds, 2000). Pharmacological doses of retinoids used in combination with other therapies are being successfully used in the treatment of various types of cancers, including NC-derived neuroblastoma and melanoma, as well as leukemia (Schenk et al., 2014; Tang and Gudas, 2011). Our findings provide an alternative viewpoint on retinoid therapy and suggest that the positive therapeutic effects of RA observed in the clinic might also be due to its ability to reverse mesenchymal transcriptional programs.

Our data indicates that RA could be an attractive therapeutic approach to both inhibit EMT and promote differentiation of cancer stem cells *in vivo*. However, retinoid resistance restricts the clinical benefits of retinoids and continues to be an issue in cancer therapy (Connolly et al., 2013; Freemantle et al., 2003). The standard use of retinoids involves direct administration into the blood stream, and its effectiveness depends on the cell’s ability to transport and/or metabolize exogenous retinoids. The TP-0903 compound identified in this study acts through a newly identified mechanism to activate RA-dependent transcription, which might alleviate retinoid toxicities and resistance. Pre-clinical studies on established RA-sensitive and -resistant tumor models will allow assessment of TP-0903 as an alternative adjuvant or co-treatment therapy to direct RA administration. To this end, TP-0903 is progressing toward a first-in-human study in 2016 after completion of repeat-dose toxicology studies in both rodent and non-rodent species, allowing a Phase 1 clinical study in patients with advanced malignancies [D.B. (Tolero Pharmaceuticals), unpublished data].

## MATERIALS AND METHODS

### Zebrafish animals and generation of the *Tg(snai1b:GFP)* transgenic lines

Zebrafish were maintained and bred as described (Westerfield, 1993) and all procedures were approved by the University of Utah Institutional Animal Care and Use Committee (IACUC#15-10011). The *Tg(sox10:mRFP)* transgenic line was described previously (Kirby et al., 2006). The 3042-bp proximal *snai1b* promoter immediately adjacent to the start ATG codon was amplified by standard PCR with primers containing *Bam*H1 (5′) and *Eco*R1 (3′) restriction-enzyme sites. The amplified fragment was sub-cloned into a modified *pEGFP-1* plasmid (Clontech) containing I-SceI meganuclease sites flanking the multiple cloning sites, and clones were verified by restriction digest and sequencing. The *pSnai1b:GFP:Isce1* plasmid was linearized with I-SceI and 10 pg of the linearized plasmid was injected into wild-type (AB strain) one-cell zebrafish embryos and monitored for transient GFP expression in the neural tube. GFP-expressing embryos were grown to produce *Tg(snai1b:GFP)* transgenic germ-line founders. The *Tg(snai1b:GFP)^zd1100^* strain expressed GFP in the same locations as endogenous *snai1b* and was used in all subsequent studies (see Fig. 1 and Fig. S1).

### RNA *in situ* hybridizations

Embryos were staged by morphological criteria as described (Kimmel et al., 1995). Whole-mount *in situ* hybridizations were carried out as described (Thisse et al., 1993) and antisense probes generated for the following probes: *cyp26a1* (Shelton et al., 2006), *krox20* (Oxtoby and Jowett, 1993) and *crestin*, *snai1b*, *dlx2a* and *mitfa* (Stewart et al., 2006). The *twist1a* cDNA was generated by one-step RT-PCR and cloned into pGEM-T Easy plasmid (Promega) using primers: Forward 5′-GCAATCTGAGCTTTTCCAGAGG-3′, Reverse 5′-ATCCTTATTTTCGCCCTTG-3′. Anti-sense *twist1a* probe was generated using T7 polymerase after linearization with *Spe*1. The *hoxb1a probe* was generated with SP6 polymerase after linearization with *Eco*RV. Embryos were imaged using a Nikon C-DSD115 microscope with an Olympus DP72 camera. Identical settings were used to obtain *in situ* images within data sets. Brightness and contrast for final images were adjusted equally across data sets using Photoshop CS4.

### Time-lapse confocal imaging

Zebrafish embryos were mounted in 35-mm glass-bottom microwell dishes with 1% low-melting-point agarose in E3 egg water. Mounted embryos were submerged in egg water or egg water containing DMSO, TP-0903, RA and/or DEAB. Time-lapse images were acquired using a Olympus Fluoview FV1200 confocal microscope and Olympus FV10-ASW v4.1 software. 10× confocal images were acquired using an Olympus UPlanSApo 10×/0.45 objective every 10-20 min and 60× confocal images were acquired with an UPlanSApo 60×/1.20W objective every 35 min.

### Chemical compounds and small-molecule screening

*Tg(snai1b:GFP)* zebrafish embryos at ∼13 hpf were treated in 12-well plates. 8-10 embryos/well were incubated at 28°C in 1 ml of egg water containing pharmacological inhibitors or 0.1-1% DMSO as control. Pharmacological inhibitors were removed after 6-12 h incubation and embryos screened for EMT and migration defects at 20-24 hpf using fluorescent light on an Olympus SZX16 microscope. TP-0903 (Mollard et al., 2011) was reconstituted to 10 mM in DMSO. All-*trans*-retinoic acid (RA) and DEAB were purchased from Sigma. Stock solutions of RA (10 mM) and DEAB (10 mM) were prepared in DMSO. All RA treatments were performed in the dark.

### Sectioning

Zebrafish embryos were sectioned using a vibratome (Leica VT1200 S) as described (Westerfield, 1993). Embryos were staged and fixed in 4% paraformaldehyde (PFA) at room temperature (RT) for 2 h. Fixed embryos were rinsed in 1× phosphate-buffer saline (PBS; pH 7.4) 3×5 min and then soaked in 0.3 M sucrose dissolved in 1× PBS overnight (O/N) at 4°C. Yolk sacs were removed with forceps before embedding embryos in 17% gelatin dissolved in 10% Hanks Saline at 42°C. Gelatin-tissue blocks were cut into 100-μm-thick sections using vibratome. Sections were then mounted on slides and imaged, or processed and stained as free-floating slices in 12-well plates.

### Immunofluorescence

Embryos were fixed in 4% PFA at RT for 2 h and sectioned as described above. Slices were rinsed 3×5 min with 0.2% Triton X-100 in PBS and blocked in 1% DMSO, 2 mg/ml BSA, 0.5% Triton X-100 and 10% normal goat serum in PBS for 2.5 h at RT. Slices were incubated in primary antibody rabbit anti-Cdh1 (1:400, GeneTex cat. # GTX125890), rabbit anti-pan-Cadherin (1:400, Sigma cat. # C3678) and mouse anti-GFP (1:400, Clontech Laboratories cat. # 632381) O/N at 4°C. Slices were again washed in 1× PBS+0.2% Triton X-100 4×15 min and blocked for 2.5 h at RT. Subsequently, slices were incubated in secondary antibody donkey anti-mouse Alexa Fluor 488 (1:400, Invitrogen cat. # A-21202), donkey anti-rabbit Alexa Fluor 568 (1:400, Invitrogen cat. # A-10042) O/N at 4°C and washed 4×15 min in PBS+0.2% Triton X-100. Processed sections were mounted on slides and confocal imaging performed using an Olympus Fluoview FV1200 confocal microscope with Olympus FV10-ASW v4.1 software. For double-fluorescent labeling, *Tg(snai1b:GFP)* embryos at the 12-somite stage were processed as above using a *snai1b* anti-sense probe (Stewart et al., 2006) that was detected with Fast Red stain (Roche cat. # 11496549001). Embryos were then processed for GFP immunofluorescence and incubated in chicken anti-GFP primary antibody (1:1000, Aves Labs cat. # GFP-1020) at 4°C overnight and secondary goat anti-chicken Alexa Fluor 488 (1:250, Invitrogen cat. # A-11039) for 1 h at RT. To image, embryos were flat-mounted on coverslips in 80% glycerol and imaged as above.

### Analyses of EMT factors in human cell lines

PANC-1 cells were seeded in 6-well plates at 1×10^6^ cells per well in 1 ml of RPMI supplemented with 10% fetal bovine serum (FBS) (Caisson Labs) and 1% penicillin/streptomycin (Caisson Labs). Cells were then treated with varying concentrations of TP-0903 and incubated at 37°C, 5% CO_2_ for 2 or 24 h. RNA extraction was performed with the NucleoSpin RNA isolation kit (Macherey-Nagel) according to the manufacturer's protocol. The iScript™ Reverse Transcription Supermix (Bio-Rad) was then used for cDNA synthesis from 1 µg of mRNA samples. The PCR reactions were run in 10-µl duplicates using commercially available SNAI1 (Snail), *TWIST1* (Twist) target and HPRT1 endogenous control primers (TaqMan), TaqMan Gene Expression Master Mix, and 60 ng of cDNA per well. RT-qPCR was performed using the 7500 Fast Real-Time PCR System (Applied Biosystems). Two-way ANOVA was used to measure statistical significance.

For analysis of protein expression, primary antibodies for SNAIL1 (cat. # 3879S) and β-ACTIN (cat. # 4967L) were purchased from Cell Signaling Technologies (Danvers, MA). Protein lysates from PANC-1 cells treated with DMSO or different doses of TP-0903 were extracted with lysis buffer (Cell Signaling) and quantified using bicinchoninic acid (BCA) protein assay (ThermoFisher). Protein lysates from the different treatment conditions were split equally and loaded on two separate sodium dodecyl sulfate polyacrylamide gel electrophoresis (SDS-PAGE) gels and transferred to polyvinylidene difluoride (PVDF) membranes for immunodetection to detect β-ACTIN (loading control) or SNAIL1. Primary antibodies were used at a dilution of 1:1000. Immunoreactive bands were detected using the Immobilon Western chemiluminescent horseradish peroxidase (HRP) substrate (EMD Millipore).

### RNA sequencing

Embryos were treated at 13 hpf (8 somites) with TP-0903 and DMSO for 1, 4 and 8 h at 28°C. 35 embryos were collected for each treatment. 4 biological replicates were sequenced for each condition. The average read count per sample prior to mapping was 20 million. 92% (s.d. 0.04) of reads aligned to the reference; 68% (s.d. 0.08) aligned to a single location in the genome. Of the uniquely aligned reads, 75% (s.d. 0.02) aligned to known gene annotations. The average Pearson correlation between replicate samples was 0.9982. RNA was harvested using Qiagen RNeasy kit (cat. # 74104). Quality of RNA was assessed using Bioanalyzer RNA 6000 Nano Chip and RNA-Seq libraries prepared using Illumina TruSeq Stranded Total RNA Sample Prep Kit with Ribo-Zero (cat. # RS-122-2401). Sequencing was performed on an Illumina HiSeq 2000 using a 50-cycle single-read sequencing v3 kit (cat. # FC-401-3002).

USeq's MakeTranscriptome (v8.8.1) application was used to create all possible splice-junction sequences for each gene, using ensemble transcript annotations. The splice junction sequences were combined with zv9 chromosome sequences and used to create a Novoalign (v2.08.01) index. Reads were aligned to the reference using Novoalign, allowing up to 50 alignments for each read. The resulting alignment file was processed with USeq's SamTranscriptomeParser application, which selects the appropriate alignment location for each read and discards repetitive alignments or alignments with low qualities. SamTranscriptomeParser also converts splice-junction alignment coordinates back to genomic space. The processed alignments were then run through USeq's DefinedRegionDifferentialSeq (DRDS) application, which counts the number of alignments to each gene. Subsequently, differential expression analysis was generated on the count data with DESeq2 using default settings and a negative binomial distribution test (Love et al., 2014). All high-throughput sequencing data sets have been submitted to Gene Expression Omnibus (GEO) and can be accessed through GEO accession number GSE72322 (http://www.ncbi.nlm.nih.gov/geo/query/acc.cgi?acc=GSE72322).
